# The Depression Conundrum and the Advantages of Uncertainty

**DOI:** 10.3389/fpsyg.2017.00939

**Published:** 2017-06-28

**Authors:** Jan E. Celie, Tom Loeys, Mattias Desmet, Paul Verhaeghe

**Affiliations:** ^1^Department of Psychoanalysis and Clinical Consulting, Faculty of Psychology, Ghent UniversityGhent, Belgium; ^2^Department of Statistics and Data Analysis, Faculty of Psychology, Ghent UniversityGhent, Belgium

**Keywords:** depression, EBTs, meta-analysis, effectiveness, quantitative research

## Abstract

According to the WHO (2012), the prevalence of unipolar depressive disorders is rising, even in those places where mental health treatments are widely available. The WHO predicts that these disorders will be the leading contributor to the global burden of disease by 2030. This sobering projection fits poorly with how psychological treatments for depression are presented in the mainstream scientific literature: as highly effective therapies, based upon a sound understanding of the causes of distress. There is a clear discrepancy between the rising prevalence figures on the one hand, and the confident claims of this effectiveness research on the other. This discrepancy prompts a set of complex interlinked questions, which we have called ‘The Depression Conundrum.’ In search of a partial answer, the aim of our study was to critically analyze five meta-analytic studies investigating the effectiveness of psychological EBTs for depression, all of which had been published in high impact factor journals. Our examination established a number of methodological and statistical shortcomings in every study. Furthermore, we argue that the meta-analytic technique is founded upon problematic assumptions. The implications of our analysis are clear: decades of quantitative research might not allow us to conclude that psychological EBTs for depression are effective. The uncertainty and questions raised by our findings might act as a catalyst to broaden the way in which depression and associated therapies are researched. In addition, it might contribute toward a more vigorous and interdisciplinary debate about how to tackle this soon-to-be global public health priority number one.

## Introduction

Several epidemiological studies have estimated that depressive disorders are increasingly prevalent in the general population around the globe (Ormel et al., 1994; Berardi et al., 1999; Demyttenaere et al., 2004; Kirsch, 2009; Moloney, 2013). As of date, the lifetime prevalence for Major Depressive Disorder (MDD) in the United States has risen to 16.6% with an economic expenditure for society of approximately $ 83.1 billion (Greenberg et al., 2003; Kessler et al., 2005). Lifetime prevalence for MDD and dysthymia in Europe are 12.8% and 4.1%, respectively, and point prevalence of MDD and dysthymia in different European countries varies between 1.1 and 3.9% (Alonso et al., 2004a,b). Overall depression accounts for 11% of disability worldwide and these disability figures are rising (Hirschfeld et al., 2000; Stewart et al., 2003). Today, it is argued that MDD is a chronic disease, prompting Murray and Lopez’s (1997) prediction that MDD will be the second overall cause of disability worldwide by the year 2020. The WHO (2012) suggests that depression is the leading cause of lost productivity due to disability. According to the same study, unipolar depressive disorders were ranked as the third leading cause of the global burden of disease in 2004 and will move into the first place by 2030. Dehue (2008) quite rightly talks about ‘*The Depression Epidemic.*’

### The Therapeutic Mastery of Depression: ‘Everybody Has Won and All Must Have Prizes’

Scores of meta-analyses investigating the effectiveness of psychotherapeutic interventions for depression have been conducted over the past decades (Dobson, 1989; Leichsenring, 2001; Westen and Morrison, 2001; Cuijpers et al., 2007a,b, 2008, 2011a,b; Ekers et al., 2008; Kirsch, 2009; Driessen et al., 2010; Huntley et al., 2012; Moloney, 2013). Butler et al. (2006) even published a systematic review of these meta-analyses. Based on these meta-analytical findings, a seemingly well-established twofold scientific consensus seems to be broadly accepted by most academics.

First, nearly all of this outcome research claims that it is now well established that all researched forms of psychotherapy are effective evidence-based treatments (EBTs) in the treatment of depressive disorders as defined in either the Diagnostic and Statistical Manual of Mental Disorders (DSM), the International Classification of Disease (ICD) or the Research Diagnostic Criteria (RDC). Substantial effects of psychological interventions compared to control conditions have been repeatedly documented over the past decades through a myriad of meta-analytic studies. Moreover, this research suggests that these EBTs arise from or lead to scientifically grounded theories explaining the origins of this condition.

Second, research claims that very different psychotherapeutic approaches have comparable benefits (Rosenzweig, 1936; Stiles et al., 1986; Bergin and Garfield, 1994). ‘Everybody has won and all must have prizes’ or the well-known ‘Dodo Bird Verdict’ was broadly introduced in the field of clinical psychology by Luborsky et al. (1975). Countless studies confirmed the Dodo Bird Verdict and so, today, a majority of researchers argue in favor of the *equivalence hypothesis*, the hypothesis that very different forms of psychotherapy are equally effective (Verhaeghe, 2005). Miller et al. (2013) suggest to bury the hatchet between different therapeutic orientations in order to be able to start focussing on the one question that hasn’t been adequately resolved yet: ‘How does psychotherapy work?’

### The Depression Conundrum

Based upon the previous section, it is reasonable to expect that effective prophylactic measures could be implemented and that in case depression *does* occur, it could be cured. Consequently, one would anticipate that prevalence figures for depression would decline. As mentioned, this is not the case. We define this apparent contradiction between the rising prevalence figures of depression on the one hand, and the claimed therapeutic mastery of this mental health condition on the other, as ‘*The Depression Conundrum.*’

We suggest four juxtaposed explanations for the ‘Depression Conundrum.’ First, due to lack of funding, prophylactic measures could not be adequately implemented while the number of possible social causal factors rises (e.g., economic imperatives, labor organization, unemployment, racism, sexism, oppression…) (WHO, 2012; Moloney, 2013). Second, people who suffer from depressive mood do not find easy access to adequate and effective treatments (Huntley et al., 2012). Third, people have great difficulty accepting their depressive condition and seeking treatment for it due to a negative representation of depression, due to a negative societal stigmatization of depression (Verhaeghe, 2002; Dehue, 2008; Moloney, 2013). Fourth, the effectiveness of EBTs might be overestimated. We will further focus on the latter.

The assumption shared by most quantitative researchers is that it is possible to measure symptomatology, treatment and outcome in a reliable and valid way through randomized controlled trials (RCTs) and subsequent meta-analysis. We will question this assumption on a meta-analytical level. We were supported in our research focus because expert-statisticians have convincingly exposed the presence of various fallacies and biases when researchers try to examine the effectiveness of treatments through quantitative meta-analytic research (Hunt, 1997; Berk and Freedman, 2001; Hedges and Pigott, 2001; Rosenthal and DiMatteo, 2001; Borenstein et al., 2009; Cooper et al., 2009; Matt and Cook, 2009).

### The Meta-analytic Technique: Strengths and Weaknesses

The term *meta-analysis* was coined by Gene Glass (1976) to indicate a more general analysis of different individual analyses (Cooper and Hedges, 2009). Meta-analytic research is widely regarded as a well-established method to sift, classify, simplify, and synthesize ostensibly inconsistent results from a corpus of studies presenting the highest levels of scientific evidence (Mann, 1994; Hunt, 1997; Rosenthal and DiMatteo, 2001; Cooper et al., 2009). Accordingly, meta-analytic findings are often used as the basis for policy-making (Mann, 1994; Rosenthal and DiMatteo, 2001; Cordray and Morphy, 2009; Greenhouse and Iyengar, 2009). Rosenthal and DiMatteo (2001) argue that the meta-analytic realm offers a number of advantages if and when conducted by researchers who have the necessary statistical expertise. Meta-analysis is now widely used in biomedicine, the behavioral sciences and the interface of the two (Mann, 1994; Rosenthal and DiMatteo, 2001). ‘This popularity has come about partly because these disciplines generate too much information to manage easily and methods are needed to synthesize that information’ (Rosenthal and DiMatteo, 2001, p 61). Reflecting upon this vindication, a couple of critical questions arise and need clarification.

First, what exactly is the scientific quality of all this information? In their search concerning the *reporting* within meta-analysis on the quality of the primary research, Orwin and Vevea (2009) did not find very encouraging statements. Light and Pillemer (1984, as cited in Cooper et al., 2009) called the reporting of the quality of primary research studies within research synthesis ‘shocking,’ Orwin and Cordray (1985, as cited in Cooper et al., 2009) used the word ‘deficient,’ while Oliver (1987, as cited in Cooper et al., 2009) used the term ‘appalling.’ Treatment regimens, therapist characteristics, patient characteristics, and methodological features are often inadequately reported within the primary research and/or inaccurately transcribed by coders (Orwin and Vevea, 2009). If both these processes are not adequately reported within the meta-analysis, the scientific quality of the findings might indeed be questionable. In *The Handbook of Research Synthesis and Meta-Analysis* (Cooper et al., 2009) – an important reference book used today by many research synthesists – it is argued by different expert-statisticians that the past decades of meta-analytic practice have amply demonstrated that primary studies *rarely* present good quality evidence.

Second, is the meta-analytic technique undeniably accurate in its information synthesis and does it generally lead to correct and replicable findings? Inductive research starts from data, quantifies these data and these quantifications must be converging toward underlying objective findings that can be – time and again – replicated, the latter being the very essence of the positivist paradigm (Carnap, 1928; Der Wiener Kreis, 1929; Popper, 1934; Störig, 1959/2000; Wittgenstein, 1961; Putnam, 1981; Vermeersch and Braeckman, 2008). A few critical reflections could be raised here about meta-analytic findings when judged according its own requirements. *The Handbook of Research Synthesis and Meta-Analysis* (Matt and Cook, 2009) warns of the numerous pitfalls, threats and limitations of the meta-analytic technique. Matt and Cook (2009) suggest there is a notable lack of control within meta-analytical studies for the threats to inferences about the existence of an association between treatment and outcome classes, especially *causal* associations. The main threats arise from: low statistical power, unreliability, restriction of range, missing effect sizes in primary studies, unreliability of coding, publication bias, bias in computing effect-sizes, lack of statistical independence and the under justified use of fixed- or random-effects models. All of these issues potentially degrade the integrity of later analyses and should be seen as serious threats to generalized inferences (Borenstein et al., 2009; Cooper et al., 2009; Matt and Cook, 2009).

Finally, Orwin and Vevea (2009) focus the attention on subjective judgment calls within meta-analysis. When Orwin and Cordray (1985, as cited in Cooper et al., 2009) reviewed the meta-analytical findings of Smith et al. (1980) – still a reference within the field of effectiveness research of psychotherapy – it was amply demonstrated that judgment problems arose in their research synthesis. Subjective judgment calls need to be made during meta-analysis and these possibly have an impact on the quality of the meta-analysis and, accordingly, on the presented effect-sizes and subsequent general findings. Coding decisions on ‘therapist experience’ or ‘length of therapy,’ for example, when not clearly or accurately reported in the primary study. Often guessing conventions are created to guess the true value of a certain variable. However, unlike pure observational error, the convention-generated errors may not balance out but consistently under- or overestimate the true value (Orwin and Vevea, 2009). Since coding decisions are often based on reporting deficiencies within the primary research, they potentially combine the possibility of bias *and* error (Orwin and Vevea, 2009). These academics argue that guessing conventions artificially deflate true variance in coded variables, diminishing the sensitivity of the analysis to detect relationships with other variables. All kinds of strategies have been developed to reduce coding error, however, based on our literature review we are inclined to assume that it is extremely difficult to entirely eliminate subjectivity within the coding process. Scientific reality might indeed be, above all, a humanized reality (Kuhn, 1970; Putnam, 1981).

Beyond doubt, the meta-analytic technique brought important scientific progress to many research areas. This technique offers a unique way to synthesize the results from a corpus of studies presenting high levels of scientific evidence. ‘Potential advantages of meta-analyses include an increase in power, an improvement in precision, the ability to answer questions not posed by individual studies, and the opportunity to settle controversies arising from conflicting claims. However, they also have the potential to mislead seriously, particularly if specific study designs, within-study biases, variation across studies and reporting biases are not carefully considered’ (Higgins and Green, 2011). In what follows, we conceptualize a non-standardized analysis of five selected meta-analytic studies, primarily based on our reading of *The Handbook of Research Synthesis and Meta-Analysis* (Cooper et al., 2009).

## Method

In our study, we hypothesize that decades of quantitative research might not have resulted in unequivocal and replicable knowledge about the effectiveness of psychotherapeutic treatments for depression. If this is the case, this could – at least partially – explain ‘The Depression Conundrum.’

### A Search on the Web of Science – Inclusion Criteria

1. We searched for recently published meta-analytical studies on the effectiveness of the psychotherapeutic treatment of depression.
2. These studies had to be published in a journal with impact factor > 2. We assume these scientific publications are the ones most consulted by both researchers and clinicians and, accordingly, that these findings represent the highest scientific and social authority.
3. Every study had to deal with a different kind of research question concerning the effectiveness of the psychotherapeutic treatment of adult depression. We sought to obtain an accurate overall impression on the effectiveness of psychotherapy for depression.

**FIGURE 1:**
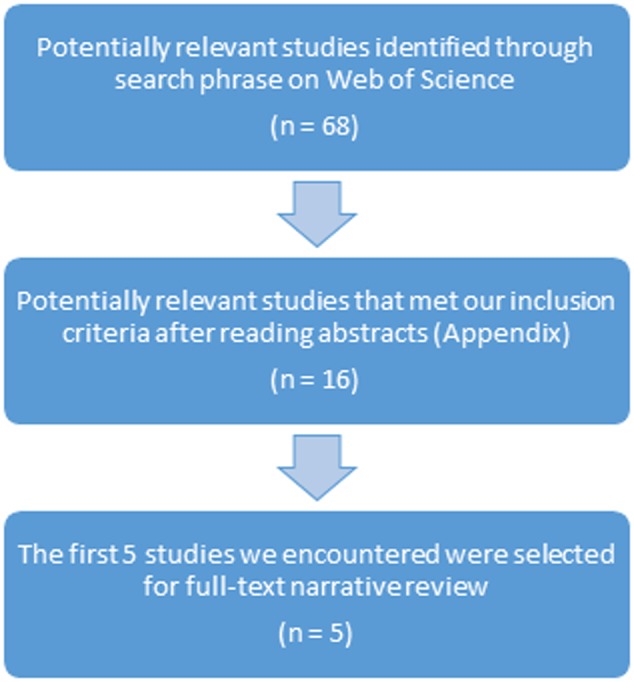
Process of selection of five meta-analytic studies for narrative review.

We conducted a search on Web of Science (**Figure 1**). The following search phrase was introduced: ‘effectiveness psychotherapy depression meta-analysis.’ We applied our inclusion criteria in our reading of the abstracts from the 68 studies yielded from the search. Then, from the 16 selected studies (**Appendix**), we chose the first five meta-analytic studies we encountered for further analysis (**Table 1**). The 11 remaining studies were also examined and we will refer to some of these.

**Table 1:** The five meta-analytic studies we analyzed in detail.

Meta-analysis	Year	Journal	Impact factor	Included studies	Main Conclusions as stated in the abstract
Bortolotti et al.	2008	General Hospital Psychiatry	2.381 High	10	*’Psychological forms of intervention are significantly linked to clinical improvement in depressive symptomatology and may be useful for supplementing usual GP care.’* (p. 293)
Barth et al.	2013	PlosMed	6.13 High	198	*’Overall our results are consistent with the notion that different psychotherapeutic interventions for depression have comparable benefits. However, the robustness of the evidence varies considerably between different psychotherapeutic treatments.’* (p. 1)
Cuijpers et al.	2010	The British Journal of Psychiatry	7.06 High	117	’*The effects of psychotherapy for adult depression seem to be overestimated considerably because of publication bias.’* (p. 173)
Wampold et al.	2011	Clinical Psychology Review	8.146 High	14	*‘Extant research on EBT versus TAU reveals that there is insufficient evidence to recommend the transportation of EBTs for anxiety and depression to routine care, particularly when routine care involves psychotherapeutic services.’* (p. 1304)
Huntley et al.	2012	The British Journal of Psychiatry	7.06 High	23	*’Group CBT confers benefit for individuals who are clinically depressed over that of usual care alone. Individually delivered CBT is more effective than group CBT immediately following treatment but after 3 months there is no evidence of difference. The quality of evidence is poor. Evidence about group psychological therapies not based on CBT is particularly limited.’* (p. 184)

### Quality Assessment Parameters

Based on *The Handbook of Research Synthesis and Meta-Analysis* (Cooper et al., 2009), we choose to focus on five quality assessment parameters in order to determine whether or not the main conclusions of our selected studies radiate scientific authority. In what follows, we argue why we choose these parameters.

First, the quality of primary research is known to have a possibly tremendous biasing effect and could therefore potentially threaten the validity of the meta-analysis (Barth et al., 2007; Matt and Cook, 2009; Valentine, 2009). As argued before, primary research studies rarely present good quality evidence (Cooper et al., 2009). High statistical power could be seen as a reliable indicator for good quality. When all primary studies included in the analysis have high statistical power, then the meta-analysis has necessarily high statistical power in the case of fixed-effects analyses or probably high statistical power in the case of random-effects analyses where heterogeneity is low (Hedges and Pigott, 2001). Hedges and Pigott (2001) clarify that statistical power of tests in fixed-effects meta-analysis depends on three parameters: sample size, effect-size and the level of statistical significance. In random-effects meta-analysis a fourth parameter is needed in order to be able to estimate statistical power: the between-studies variance component (Maxwell and Delaney, 1990; Diggle et al., 1994). It should be noted that sample size within meta-analysis has two components: the within-study sample size of the different primary research studies *and* the number of studies included in the meta-analysis. We were particularly cautious concerning meta-analyses of smaller numbers of studies or meta-analyses dealing with research questions where effects were expected to be small. In both cases, meta-analyses do not have necessarily high statistical power (Hedges and Pigott, 2001). Furthermore, the exclusion of individual studies with low statistical power provides some protection against the effects of publication bias (Kraemer et al., 1998). If researchers failed to report the statistical power of the primary research studies, we assumed they perchance included individual studies with low statistical power. In the present case, we argue that the statistical power of the meta-analysis could possibly be low and we suggest that the findings of these studies should be looked at with prudence, particularly if the number of the included primary studies was low, if effects were expected to be small or in the case of a random-effects meta-analysis where heterogeneity was moderate to high. The robustness of the findings of such studies is questionable. Policy-making based on such findings is problematic.

Second, we focused on whether or not heterogeneity-testing (e.g., chi-square test) was executed and whether or not the inconsistency value I^2^ was calculated to explore clinical and methodological heterogeneity between the different studies included in the meta-analysis. Moderate (I^2^ = 50%) to high (I^2^ = 75%) degrees of heterogeneity indicate a lower scientific weight of the findings (Higgins et al., 2002, 2003; Cooper et al., 2009). Heterogeneity should be interpreted as variability not likely due to sampling error, but due to true differences among the studies. We argue that when heterogeneity-testing was not performed or when the degree of heterogeneity was moderate to high, the findings should be looked at with caution. The sturdiness of such findings is genuinely questionable and policy-making based on such findings is precarious (Higgins et al., 2002, 2003).

Third, we investigated whether or not sensitivity analyses were conducted to further test the cogency of the obtained results. Greenhouse and Iyengar (2009) argue that – given the prominent role meta-analysis plays today in policy-making – the need for sensitivity analysis has never been greater. Sensitivity analyses could consist, for example, of excluding studies with noticeable outliers in the statistical analysis because these might distort the overall results. Multiple comparisons – who are not independent of each other – may result in an artificial reduction of heterogeneity and so it is important to conduct additional analyses in which only one comparison per study is included (Cuijpers et al., 2010).

Fourth, we argued in an earlier section that failing to examine and control for a number of other validity threats potentially degrades the integrity of later analyses and should be seen as a serious threat to generalized inferences to broader classes or universes. We investigated whether validity threats were explored.

Finally, based on the reading of Putnam (1981), Elkin (1999), Vermeersch and Braeckman (2008), Cooper et al. (2009), Miller et al. (2013), and Moloney (2013) we were also particularly interested in whether or not *causal* relationships were established between therapeutic techniques or interventions and the presumed specific effects they have. We realize that this final parameter could easily be disqualified or regarded as highly problematic. Many researchers would argue that meta-analysis is not designed to establish causality. Moreover, *causal claims* are usually not made in meta-analytic studies. However, causal relationships are nearly always *suggested* in the abstracts of meta-analytic research (e.g., Bortolotti et al., 2008, p. 293). Accordingly, we argue it is essential to specifically put forward the predicament concerning causality. How can we be sure that improvement is due to the treatment itself, rather than other variables such as the contributions made by the client, spontaneous recovery, the individual therapist’s personal characteristics, the amount of training, the supervision model used, or any other of a multitude of variables? Putting the question forward ‘Were causal relationships established?’ is indispensable within effectiveness research, even though we realize the answer to this question is invariably ‘No.’

## Results

We first present a concise appreciation about whether or not our five quality assessment parameters were examined by the five different research teams (**Table 2**). The sign (^∗^) means that the notion ‘Yes’ (examined) was found flawed or inadequate after further clarification within our more detailed individual review (e.g., lack of data when trying to assess quality of primary research, heterogeneity was tested and reported as ‘high’). Just like the answer ‘No,’ the sign (^∗^) thus represents a negative evaluation from our point of view. **Table 2** shows a total of 19 negative evaluations out of 25.

**Table 2:** Where the five quality assessment parameters examined?

	Quality Primary	Heterogeneity	Sensitivity analysis	Other validity	Causal relations
Study	Research examined?	testing executed?	executed?	threats examined?	established?
Bortolotti et al., 2008	Yes (^∗^)	Yes (^∗^)	Yes (^∗^)	No	No
Barth et al., 2013	Yes (^∗^)	Yes	Yes	No	No
Cuijpers et al., 2010	No	Yes	Yes	No	No
Wampold et al., 2011	Yes (^∗^)	Yes	No	No	No
Huntley et al., 2012	Yes (^∗^)	Yes (^∗^)	Yes	No	No

*Yes = examined. No = not examined. The sign (^∗^) means that the notion ‘Yes’ was found flawed or inadequate after further clarification within our more detailed individual review (e.g., lack of data when trying to assess quality of primary research, heterogeneity was tested and reported as ‘high’). Just like the answer ‘No,’ the sign (^∗^) thus represents a negative evaluation from our point of view. **Table 2** shows a total of 19 negative evaluations out of 25.*

### Review 1: Bortolotti et al. (2008)

*‘Psychological forms of intervention are significantly linked to clinical improvement in depressive symptomatology and may be useful for supplementing usual GP care.’* (p. 293)

This meta-analytic study is one of the few available examining the effectiveness of the psychotherapeutic treatment of depression compared to usual general practitioner (GP) or compared to antidepressant medication within primary care settings. We will focus on the first comparison. This publication starts in an encouraging manner. First, the authors excluded studies prior to 1995 because in more recent years several methodological limitations from earlier studies have been overcome (Brown and Schulberg, 1995). Next, the authors assessed the methodological quality of the included 10 trials using the Cochrane Collaboration on Depression Anxiety and Neurosis Quality Rating Scale (Moncrieff et al., 2001). Regrettably however, neither the statistical power of the individual studies nor the statistical power of the meta-analysis were reported, so we assume the authors might have included individual studies with low statistical power. This was particularly problematic given the small number of individual studies involved in this meta-analysis and our previous observations in that regard.

The main analysis showed greater effectiveness of psychological interventions over usual GP care in both short term [standardized mean difference (SMD) = -0.42, 95% CI)] and long term [SMD = -0.30, 95% CI]. Both the fixed-effects analysis and the random-effects analysis showed similar results. These overall effect-sizes for both the short-term comparison and the long-term comparison were small effects, according to Cohen’s criteria (Cohen, 1988). Because of the thoroughness of GP care provided in the examined trials, the authors suggest that these effects might be actually greater in everyday clinical practice.

Where the heterogeneity test was not significant in the short term, this test showed a high degree of heterogeneity and inconsistency in the long term (I^2^ = 70.9%) and the authors point out that they were unable to clarify the reason for this. One sensitivity analysis, excluding three studies based on their inadequate quality rating scores and/or high attrition rates increased somewhat the overall short-term estimate, but there was only a minor difference in the long-term effect-size estimates and the I^2^-value remained high for the long-term comparison. Possible publication bias was not examined, nor were any of the other validity threats we mentioned earlier explicitly identified and ruled out. The selected studies had relatively small sample sizes and high attrition rates. Causal relationships between therapeutic actions and outcome measures were not established within the primary studies. The authors conclude that, mainly due to sample characteristics, their findings could not be generalized to male, child, adolescent or elderly patients, nor could these results be generalized to non-Caucasian patients or depressed patients with substance abuse disorders or with general medical conditions.

In summary, we acknowledge the fact that the many shortcomings of this study were relatively well presented and documented within the “Discussion” Section. However, applying our quality assessment criteria, we fail to understand the conclusion as stated in the abstract. We deem that this study offers no solid scientific evidence for this kind of generalized statement. Essentially, this study shows that researchers very often are required to make do with what they have. We would not advise policy-makers to take this study into account while reflecting upon and making decisions about future policy.

### Review 2: Barth et al. (2013)

*‘Overall our results are consistent with the notion that different psychotherapeutic interventions for depression have comparable benefits. However, the robustness of the evidence varies considerably between different psychotherapeutic treatments.’* (p. 1)

The conclusions of this study reflect – albeit not perfectly – the above mentioned ‘Dodo Bird Verdict’ (Luborsky et al., 1975). Within this study, Barth et al. (2013) used a fairly new methodology – network meta-analysis – to study the comparative effectiveness of seven psychotherapeutic interventions for depression. The important benefit of this novel technique is that it allows a comparison of *all* conditions in the connected network of all the included studies: direct comparisons within the same trial and indirect comparisons across all trials. However, as the authors point out, it should be mentioned that network meta-analysis makes the questionable assumption that all included trials originate from the same homogeneous population. A second debatable assumption is made within network meta-analysis, namely, that different treatments each have their own specific rationale and procedure. This allows researchers to group the different treatments and represent them each as one knot in the network. Putting ‘brand names’ on different forms of psychotherapy is not entirely self-evident. Shedler (2010) argues – from a psychodynamic perspective – that the active ingredients of other therapies include techniques that have long been central to psychodynamic theory and practice. Miller et al. (2013) – taking a different perspective – claim that we do not know how psychotherapy works, that we do not have a clear understanding of its active ingredients, specific rationales and procedures.

A total of 198 primary studies were included in the network, representing 433 conditions, seven different types of psychotherapy and 15,118 adults with depression. Heterogeneity within the network meta-analysis was low and there was no evidence that direct and indirect estimates were inconsistent. As the authors notice, all of this suggested very good interpretability. Moderate to large effects were found for all seven forms of psychotherapy when compared to the waitlisted subjects. When these interventions were compared to usual care or placebo condition (psychological or pharmaceutical), the researchers found small to moderate effects, except for social skills training. Small or no differences were found when the different interventions were compared with each other and there was no significant difference in effect size when individually delivered therapy was compared with group therapy or internet-based interventions. Indeed, everybody seemed to have won and all seemed to deserve a prize.

However, the authors then put their rather promising initial findings into a more balanced perspective. Treatment effects were smaller when outcome was assessed through self-report measures, blinded observers or within studies where randomization was not adequately concealed. Smaller and less stringently designed studies found larger benefits of psychotherapy and since 162 studies (out of 198 studies) involved in this network meta-analysis had a small sample size (<25), stepwise restriction was implemented. Stepwise restriction to only studies with moderate sample size (25 to <50) and large sample size (>50) reduced the effect-sizes of all treatments. Stepwise restriction to only studies with large sample size – because of their higher study quality the more accurate analysis within the network – showed moderate effects for only three interventions when compared to waitlist. The reason for this was that stepwise restriction reduced the number of interventions that could be adequately represented in the network. Moreover, most primary studies in this network meta-analysis were conducted in Western countries (58% in the United States) and this could be considered as a serious threat to generalized inferences to broader classes or universes. Of all the studies, 70% investigated cognitive-behavioral interventions while, for example, only 4% of the studies investigated social skills training. Finally, causal relationships between specific therapeutic actions and outcome measures were not established and many validity threats we mentioned before were neither identified nor controlled for in this study.

The authors of this study invite for critical reflection through their exhaustive methodological set-up, through the way the initial findings were put into perspective and through exposing accurately the limitations of their study. We believe the authors would agree with the following additional conclusion: this study primarily shows that a number of distressing issues (supra) surround meta-analytic effectiveness research. This is why we do not agree with the conclusion as stated in the abstract. The findings of this study could be considered as possibly *indicative*, but these findings cannot be presented to the public at large or to policy makers as if they represent a well-grounded scientific consensus.

### Review 3: Cuijpers et al. (2010)

‘*The effects of psychotherapy for adult depression seem to be overestimated considerably because of publication bias.’* (p. 173)

Publication bias is often seen as a serious menace to the validity of a meta-analytic study (Rosenthal, 1979; Rothstein et al., 2005; Sterne and Egger, 2005; Sutton, 2009). This bias should be interpreted as a mixture of selective publication and selective reporting of outcomes. The goal of this study was to examine indicators of publication bias and to calculate effect sizes that were adjusted for publication bias in order to determine whether or not these adjusted effect sizes differed significantly from the initially presented overall effect sizes. The selection of 117 controlled studies (9537 participants) in which 175 psychotherapeutic treatment conditions were compared with different control conditions, as well as the statistical analyses of the collected data, were carefully carried out. However, the statistical power of the primary research studies was not explicitly reported, many other validity threats (supra) were not identified and controlled for in this study and causal relations between specific therapeutic actions and their presumed outcomes were not explored in this study.

The overall effect size of all 175 comparisons was *d* = 0.67 (95% CI), which is considered a medium effect (Cohen, 1988). This effect size should be interpreted with caution since heterogeneity was high (I^2^ = 70.27). Moreover, the funnel plot, first without and then with the imputed studies, showed that smaller studies with lower effect sizes were missing, which indicated the existence of publication bias. Both the Begg and Mazumbar’s test and the Egger’s test resulted in highly significant indicators of publication bias (*P* < 0.001). Adjustment for publication bias according to Duval and Tweedie’s trim and fill procedure resulted in a considerable decrease of the overall effect size to *d* = 0.42 (95% CI). Examining the possible influence of outliers, a new analysis was performed in which all effect sizes of *d* = 1.5 or larger were removed. This resulted in an overall mean effect size of *d* = 0.51 (95% CI). After correcting for publication bias, again, the overall effect size dropped considerably to *d* = 0.39 (95% CI). Furthermore, since multiple comparisons are not independent from each other, the authors executed analyses in which they included just one comparison per study (comparison with the largest effect size and comparison with the lowest effect size). All indicators of publication bias remained significant (*P* < 0.001). Further sensitivity analyses showed that these indicators remained significant for most subgroups of studies. Because more than half of the comparisons examined CBT, a separate analysis was conducted for this type of treatment. By and large, the results were very similar. Unfortunately the authors failed to mention the degree of heterogeneity for this subgroup. Cuijpers et al. warn that tests for publication bias do not provide direct evidence of such bias and that no statistical imputation method can indeed recover the ‘missing truth.’ Furthermore, they point out that there are certain weaknesses within the statistical tests used to assess publication bias such as strong dependence on certain assumptions, a tendency for low statistical power of some of these tests or the fact that the algorithm for detecting asymmetry can be influenced by only one or two aberrant studies.

However, given the highly significant indicators of funnel plot asymmetry and based on the ‘sobering lesson’ to be learned from Rosenthal’s’ (1979) eminent article, we agree with two major conclusions of this research. First, it is very likely that meta-analytic studies overestimate the true effect size of psychotherapy for adult depression due to publication bias. Second, it seems that this effectiveness research is no freer from publication bias than the research on the pharmaceutical treatment of depression as described by Dehue (2008), Turner et al. (2008), or Kirsch (2009). As Cuijpers et al. point out, and as is recently suggested by Moloney (2013), psychological treatments have an economic incentive to make the most positive case possible. Psychological treatments that are able to present large effects lead to prestige, power, subsequent lucrative workshop fees and higher session fees.

What does the impact of controlling for just *one* validity threat tell us about the notion ‘evidence-based treatments’? Unraveling ‘The Depression Conundrum’ – at least partially – might be easier than initially anticipated. In fact, after reviewing this third meta-analytic study, the following question could be put forward: ‘Might the therapeutic mastery of depression be an illusionary proposition?’

### Review 4: Wampold et al. (2011)

*‘Extant research on EBT versus TAU reveals that there is insufficient evidence to recommend the transportation of EBTs for anxiety and depression to routine care, particularly when routine care involves psychotherapeutic services.’* (p. 1304)

Within this meta-analytic study the relative efficacy of EBTs when compared to treatment as usual (TAU) was examined through direct comparisons while, at the same time, examining possible confounds such as heterogeneity within the TAU conditions (e.g., TAU with or without psychotherapeutic services) or the specialized training and expertise within EBT conditions. The researchers hypothesized that EBTs would be superior to TAU in the treatment of anxiety and depression in an adult population, but that confounds would moderate this effect. We could argue that some subjective choices made by the research team were quite arbitrary (e.g., the six point rating scale to assess researchers allegiance), however, the methodological set-up of this meta-analytic study deserves recognition. Unfortunately, this study showed primarily what we noted before: researchers are very often required to make do with what they have.

Only 14 studies met the inclusion criteria of this meta-analysis, much of the information regarding the quality of the primary research (e.g., treatment dose, training, supervision, adherence checks), used to assess the overall comparison of EBT versus TAU, was unreported. **Table 1** of this publication clearly shows this distressing fact: in roughly half of the coding sections, the researchers indicated: information not available. When information *was* reported, the authors point out that the design explicitly favored EBTs. In only three of the included studies, TAU involved psychotherapeutic treatment, but, again, within these studies the EBT condition was favored (e.g., therapists received additional training and supervision). We found a general lack of data in the first part of the results section and so, the aim of directly comparing EBT versus TAU while examining possible confounds was going to be difficult.

The meta-analysis showed that the overall effect for EBTs versus TAU was *d* = 0.45 which was significantly greater than zero (*p* < 0.01) and which represents a significant small to medium effect in favor of EBTs (Cohen, 1988). However, inconsistency was moderate (I^2^ = 58%) which provides evidence that the variability among the primary studies was not likely due to sampling error, but due to true differences among the studies. The authors were not able to model how several design confounds would account for this variability. For the between groups test, the mean effect for studies in which TAU was unlikely to be a psychotherapeutic intervention was *d* = 0.50 in favor of EBTs, which was significantly greater than zero (*k* = 9, *p* < 0.01). The mean effect size for studies in which TAU clearly comprised psychotherapeutic interventions was *d* = 0.33 in favor of EBTs, which was not significantly different from zero (*k* = 3, *p* = 0.06). The difference between these two effect sizes was not significantly different from zero (df = 1, *p* = 0.46).

The authors suggest that strong conclusions and important recommendations were not possible. We fail to understand why they argue in the closing paragraph that ‘there does appear to be evidence that implementing EBTs into routine care that does not involve psychotherapy would improve the quality of care.’ Few studies were included in this meta-analysis. Those that were included failed to report crucial information and the design, time and again, explicitly favored EBTs. Statistical power of the primary studies was not reported, heterogeneity was moderate, final results were not controlled for a number of validity threats (supra). Finally, causal relationships between specific therapeutic actions and outcome were not explored.

Indeed, contrary to what one would expect (cf. the alleged effectiveness of EBTs for depression), this study offers insufficient evidence for the transportation of EBTs to routine care. There were not enough data available and, consequently, meaningful statistical analyses of direct comparisons were impossible. Essentially, this study offers valid arguments for the following conclusion: good quality primary research is hard to find and ‘no meta-analysis can ever rise above the quality of the data upon which it depends’ (Moloney, 2013, p 92).

### Review 5: Huntley et al. (2012)

*‘Group CBT confers benefit for individuals who are clinically depressed over that of usual care alone. Individually delivered CBT is more effective than group CBT immediately following treatment but after 3 months there is no evidence of difference. The quality of evidence is poor. Evidence about group psychological therapies not based on CBT is particularly limited.’* (p. 184)

This publication starts by indicating that waiting lists are long, that resources are limited, that there is a paucity of evidence concerning the effectiveness of group CBT and that group therapies factually treat more patients at the same time and could therefore be more cost-effective.

Twenty-three original RCTs were included in this analysis. Patients were adults of either gender with a primary diagnosis of depression. Group CBT was defined as any form of psychological intervention of three or more participants. Post-treatment outcome was assessed as well as short-term outcome (>1 week to 3 months inclusive) and medium to long-term outcome (>3 months). The secondary outcome measure was cost-effectiveness. The quality of the included studies was assessed using the Cochrane Collaboration’s domain-based evaluation tool for assessing risk of bias (Higgins and Green, 2006, 2011). Because of the nature of the studies that met the inclusion criteria, the authors decided to mainly focus on two comparisons: group CBT versus usual care alone (14 studies, 1217 participants) and group CBT versus individually delivered CBT (7 studies, 211 participants).

Many of the 21 studies involved in the two main comparisons had several methodological weaknesses and showed a general lack of sufficient information concerning for example allocation concealment or individuals who had dropped out. A major methodological problem was the small sample size of many studies involved: ‘Ten studies (43%) had less than 15 participants in the intervention study arm(s), eight studies (35%) had 16–50 participants per arm and only five studies (22%) had 51 or more participants per arm’ (p 186). There was no information provided about the statistical power of the primary research studies nor about the statistical power of the meta-analysis. Based on the aforementioned evaluation tool, Huntley et al. (2012) indicated that there was a considerable risk of bias within their study. Causal relationships between specific therapeutic actions and their presumed specific effect(s) were not established and a majority of studies allowed patients within the main comparisons to take concomitant antidepressant medication.

When comparing group CBT to usual care alone, the authors found immediately post-treatment a significant medium treatment effect in favor of group CBT (14 studies, SMD = -0.55). Further well-advised sensitivity analysis presented no difference to these findings. Only three studies provided data for short-term and medium to long-term follow-up and these studies also showed a medium effect of group CBT over usual care alone (SMD = -0.47 and SMD = -0.47, respectively). However, confidence intervals were wide and there was considerable heterogeneity between effect sizes. When comparing group CBT to individually delivered CBT, the authors found immediately post-treatment a small treatment effect in favor of individual CBT (seven studies, SMD = 0.38). No difference in treatment effect was found between the two conditions on short term and medium to long term follow-up. As far as the secondary outcome measure – cost-effectiveness – was concerned, again, the lack of relevant data was notable (e.g., two studies). There were indications that group therapies are marginally more expensive than usual care alone and more cost-effective than individually delivered treatment.

The authors themselves consider the quality of their evidence as ‘poor.’ Given the numerous shortcomings of this study, we argue that the quality of evidence of this study might be considered ‘extremely poor.’ As such, we fail to understand why, in the closing paragraph of this study, Huntley et al. (2012) indicate that the evidence to support the development of group-based interventions is ‘limited but auspicious.’ This meta-analysis offers no auspicious evidence in favor of such development, let alone firm scientific evidence. What this study might indicate is the importance of belonging to and sharing with a group of like-minded individuals. The latter resonates with basic logical reasoning and common sense.

## Discussion

### The Issue of Uncertainty

In our introduction, we identified a puzzling situation surrounding depressive mood. On the one hand, we observed that the academic community suggests that we have a considerable understanding of the causes of depression and that we have different psychological EBTs to successfully treat this condition. On the other hand, we noted that worldwide prevalence figures for depression are rising and that the WHO (2012) predicts that unipolar depressive disorders will be the leading disorder in the global burden of disease by 2030. We called this apparent contradiction ‘The Depression Conundrum’ and we proposed four juxtaposed explanations. In this paper, we focused on one of these explanations. We examined the methodological quality of the effectiveness research on the subject-matter.

We analyzed five recently published meta-analytic studies representing 362 RCTs. We established a number of methodological and statistical shortcomings in every study. **Table 2** shows a total of 19 negative evaluations out of 25, meaning that we established in our more detailed review that crucial parameters were either not examined or – when examined – found flawed (e.g., low statistical power, high heterogeneity, lack of data). We argue that unraveling ‘The Depression Conundrum’ – at least partially – might be easier than initially anticipated because the implications of our analysis are clear: decades of quantitative research might not allow us to conclude that psychological EBTs for depression are effective.

Following Wood and Eagly’s (2009) treatise, we deem that our critical reflections place the field of research synthesis in a state of underlying uncertainty. Even though these expert-statisticians argue that research synthesis *rarely* provides definitive answers to the theoretical or empirical questions that inspired the investigation, the question ‘How to deal with this issue of uncertainty?’ needs to be addressed. Do we need more vigorously executed RCTs and meta-analyses? Do we need a more qualitative research approach? In what follows, we argue there is something to be said for both approaches.

### The Possibility of Gain through Additional Quantitative Research

Uncertainty suggests a need for additional research and well-supported findings and theories (Wood and Eagly, 2009). The latter should not be treated as a rhetorical device. ‘Findings that are homogeneous given adequate power and an appropriate range of conditions suggest an empirical result that can be accepted with some certainty’ (p. 457). Following this disquisition, we suggest to focus on a network meta-analysis with the following characteristics. A substantial number of high quality RCT’s examining the effectiveness of different psychotherapeutic interventions should be included. Each RCT should show the following characteristics: large sample size, high statistical power, blinded outcome assessment, adequately concealed randomization and the establishment of causal relationships between specific therapeutic actions and outcome. The subsequent meta-analysis should show the following features: high statistical power, low heterogeneity and low inconsistency. Final results should be controlled for all the validity threats and potential biases we discussed earlier. The possible gain of this approach would be that through such design we would be able to actually collect homogeneous findings, meeting the highest levels of scientific evidence. Unfortunately, this approach – however, valuable it might be – will take a huge amount of time and funding.

### The Downside Risk of Additional Quantitative Research

Berk and Freedman (2001) argue – based upon their own expertise and upon a multitude of papers by other expert-statisticians – that some of the key assumptions of the meta-analytic technique are highly debatable or worse: ‘esoteric as to be unfathomable and hence immune for rational consideration’ (p. 13).

The key assumption as if subjects are drawn at random from populations is considered gratuitous. The assumptions as if $Y_{\mathrm{ij}}^{E}$(experimentals) and $Y_{\mathrm{ij}}^{C}$ (controls) are independent and identically distributed, that these have a common expectation $\mu_{i}^{E}$ and $\mu_{i}^{C}$ and that the variances $\sigma_{i}^{2}$of both the experimental and the control condition are equal, are regarded by these expert-statisticians as ‘phantasmagorical.’ Berk and Freedman (2001) reason that different studies within a meta-analysis cannot be independent of one another because of an underlying ‘social dependence.’ ‘Investigators are trained in similar ways, read the same papers, talk to one another, write proposals for funding to the same agencies, and publish the findings after peer review. Earlier studies beget later studies, just as each generation of Ph.D. students trains the next.’ (p. 12). All these cardinal assumptions within meta-analysis are considered as often ‘pleasing,’ yet ‘illusory’ (Berk and Freedman, 2001, p. 12). It could be argued that statistical models are often extended in one way or another in an attempt to evade certain statistical and methodological problems (e.g., random effects models). However, Berk and Freedman (2001) clarify that these extensions do not in any way make these models substantially more believable. They conclude that meta-analysis would be an excellent method for research synthesis *if* the assumptions held.

We mentioned earlier that meta-analysis has the potential to mislead seriously because of methodological and statistical flaws (Higgins and Green, 2006, 2011; Cooper et al., 2009). If we combine this with the critical reflections made by Berk and Freedman (2001) concerning the underlying assumptions, we infer that there is a downside risk to the call for additional quantitative research.

### The Constructivist-Interpretative Stance within Qualitative Research

A second scientific approach departs from a very different reasoning. Within this line of thought, arguments are developed to adopt the idea that quantitative research might not be able to entirely capture the essence of depressive mood (e.g., symptom assessment) or the effectiveness of different EBTs (Elkin, 1999; Verhaeghe, 2002; Dehue, 2008; WHO, 2012; Moloney, 2013).

Assuming that impoverished or pathogenic environments or contexts trigger depressive mood (WHO, 2009, 2012; Pilgrim and Rogers, 2010; Moloney, 2013), we suggest to make far greater use of qualitative research, designed to explore and study all kinds of phenomena within their specific contexts while at the same time offering the possibility to map the prevailing socio-economic and political hegemonic discourses. The multiplicity of qualitative inquiry (e.g., narrative research, discourse analysis, interpretative phenomenological research) epitomizes the potential strength of this approach (Potter and Wetherell, 1987; Burman and Parker, 1993; Marecek, 2003; Madill and Gough, 2008). Qualitative research departs from the premise that humans are intentional and meaning-making agents. It thereby asks ‘how’-questions instead of ‘why’-questions. For example, in regard to the experience of distress and of mental health treatments, qualitative research would aim at exploring in detail the contexts in which depressed people live and the particular functionality of a symptom (Vanheule, 2015). A global research focus on the similarities between these contexts might turn out to be of importance.

Even though not well represented in the mainstream scientific literature, *meta-syntheses* – the qualitative equivalent for meta-analyses – have already delivered some promising new insights that would be difficult to obtain through quantitative research. For example: computerized therapy for depression could ameliorate considerably through personalization and sensitization of content to individual users, recognizing the need for users to experience a sense of ‘self’ in the treatment which is at present lacking (Knowles et al., 2014). Also, the finding that traditional masculinity values could serve as barriers but equally as facilitators in the development of coping strategies in depressed men (Krumm et al., 2017).

In an attempt to deal more effectively with the rising prevalence figures for depression, this approach could potentially lead to different kinds of research questions asked concerning the nature and causes of depressive mood, the types of treatment on offer for it, and their effectiveness. The constructivist-interpretative stance within qualitative research allows researchers to expose that there is not ‘one’ world or one ‘objective reality,’ but different *perspectives* on the world (Putnam, 1981; Potter and Wetherell, 1987; Burman and Parker, 1993; Marecek, 2003; Madill and Gough, 2008). It should be noted that a common critique of the epistemology of qualitative research as solely inductive is not correct. In fact, qualitative research is often driven by theory (Marecek, 2003; Vanheule, 2015). Likewise, it should be noted that this qualitative approach will also take a huge amount of time and funding.

### Limitations

We included a relatively small number of meta-analytic studies in this article, yet we did not refrain from making some critical statements. This could be seen as an important limitation. In addition, given the scope of this article, we could not further explore the vast impact of different validity threats to meta-analytic inferences as described in Cooper et al. (2009). We realize we did not provide many specific new angles from which the academic community could further explore depressive mood. As might be clear, we are currently reflecting upon meaningful alternatives from different interdisciplinary angles.

## Conclusion: the Advantages of Uncertainty

Following Wood and Eagly’s (2009) reasoning, we conclude that the implications of our critical reflections place the field of clinical psychology in a state of underlying uncertainty. However, this uncertainty has mainly two advantages. First, this uncertainty is an indirect plea for scientific humility within the field of clinical psychology, meaning that the outcome of quantitative research concerning the effectiveness of the psychological treatment of depression could be considered as indicative, but not as representing a well-established scientific consensus. Second, uncertainty stimulates new lines of thought and might therefore be considered as an indirect plea for a more qualitative research approach to better explore singular contexts, working-mechanisms, the functionality of a symptom, treatment and effectiveness.

The very nature of scientific psychology discourse surrounding depression throughout the past decades may well have led to tunnel vision and to theoretical immobility. We argue in favor of a new and vigorous scientific debate concerning depression. A platform where different kinds of research questions could be raised in an attempt to deal with this soon-to-be public health priority number one. Nothing numbs the human mind as fundamentally as hearing the same familiar words, slogans or scientific statements over and over again (Feyerabend, 1975).

## Author Contributions

All authors made substantial contributions to both the conception and design of the work, the acquisition, analysis and interpretation of data. JC was the principal author of the paper. TL focussed on methodological and statistical analysis and inferences, while MD and PV focussed on methodological, theoretical, and ethical issues. All authors approved to submit this manuscript and are accountable for all aspects of the work.

## Conflict of Interest Statement

The authors declare that the research was conducted in the absence of any commercial or financial relationships that could be construed as a potential conflict of interest.

## APPENDIX

### List of the 16 Studies Meeting Our Inclusion Criteria

1. Bortolotti, B., Menchetti, M., Bellini, F., Montaguti, M. B., and Berardi, D. (2008). Psychological interventions for major depression in primary care: a meta-analytic review of randomized controlled trials. *Gen. Hosp. Psychiatry* 30, 293–302.
2. Barth, J., Munder, T., Gerger, H., Nüesch, E., Trelle, S., Znoj, H., et al. (2013). Comparative efficacy of seven psychotherapeutic interventions for patients with depression: a network meta-analysis. *PLoS Med*. 10:e1001454. doi: 10.1371/journal.pmed.1001454
3. Cuijpers, P., Smit, F., Bohlmeijer, E., Hollon, S. D., and Andersson, G. (2010). Efficacy of cognitive-behavioral therapy and other psychological treatments for adult depression: a meta-analytic study of publication bias. *Br. J. Psychiatry* 196, 173–178.
4. Wampold, B. E., Budge, S. L., Laska, K. M., Del Re, A. C., Baardseth, T. P., Fluckinger, C., et al. (2011) Evidence-based treatments for depression and anxiety versus treatment-as-usual: a meta-analysis of direct comparisons. *Clin. Psychol. Rev.* 31, 1304–1312.
5. Huntley, A. L., Araya, R., and Salisbury, C. (2012). Group psychological therapies for depression in the community: systematic review and meta-analysis. *Br. J. Psychiatry* 200, 184–190.
6. Cuijpers, P., van Straten A., and Warmerdam, L. (2007). Behavioral activation treatments of depression: a meta-analysis. *Clin. Psychol. Rev.* 27, 318–326.
7. Saman, Z., Brealy, S., and Gilbody, S. (2011). The effectiveness of behavioural therapy for the treatment of depression in older adults: a meta-analysis. *Int. J. Geriatr. Psychiatry* 26, 1211–1220.
8. Ebrahim, S., Montoya, L., Truong, W., Hsu, S., Kamal el Din, M., et al. (2012). Effectiveness of cognitive behavioral therapy for depression in patients receiving disability benefits: a systematic review and individual patient data meta-analysis. *PLoS ONE* 7:e50202. doi: 10.1371/journal.pone.0050202
9. Mazzucchelli, T., Kane, R., and Rees, C. (2009). Behavioral activation treatments for depression in adults: a meta-analysis and review. *Clin. Psychol.* 16, 383–411.
10. Cuijpers, P., van Straten, A., and Warmerdam, L. (2007). Problem solving therapies for depression: A meta-analysis. *Eur. Psychiatry* 22, 9–15.
11. Leichsenring, F., and Rabung, S. (2008). Effectiveness of long-term psychodynamic psychotherapy: a meta-analysis. *JAMA* 300, 1551–1665.
12. Cuijpers, P., Geraedts, A. S., van Oppen, P., Andersson, G., Markowitz, J. C., et al. (2011). Interpersonal psychotherapy for depression: a meta-analysis. *Am. J. Psychiatry* 168, 581–592.
13. Driessen, E., Cuijpers, P., de Maat, S. G., Abbass, A. A., de Jonghe, F., et al. (2010). The efficacy of short-term psychodynamic psychotherapy for depression: a meta-analysis. *Clin. Psychol. Rev.* 30, 25–36.
14. Westen, D., and Morrison, K. (2001). A multidimensional meta-analysis of treatments for depression, panic, and generalized anxiety disorder: an empirical examination of the status of empirically supported therapies. *J. Consult. Clin. Psychol.* 69, 875–899.
15. Stalder-Lüthy, F., Messerli-Bürgy, N., Hofer, H., Frischknecht, E., Znoj, H., and Barth, J. (2013). Effect of psychological interventions on depressive symptoms in long-term rehabilitation after an acquired brain injury: a systematic review and meta-analysis. *Arch. Phys. Med. Rehabil.* 94, 1386–1397.
16. Foroushani, P. S., Scheider, J., and Assareh, N. (2011). Meta-review of the effectiveness of computerised CBT in treating depression. *BMC Psychiatry* 11, 131.

## References

[B1] Alonso J. Angermeyer M. C. Bernert S. Bruffaerts R. Brugha T. S. Bryson H. (2004a). Prevalence of mental disorders in Europe: results from the European study of epidemiology of mental disorders (ESEMeD) project. *Acta Psychiatr. Scand. Suppl.* 109 21–27. 10.1111/j.1600-0047.2004.00327.x 15128384

[B2] Alonso J. Angermeyer M. C. Bernert S. Bruffaerts R. Brugha T. S. Bryson H. (2004b). 12-Month comorbidity patterns and associated factors in Europe: results from the European Study of Epidemiology of Mental Disorders (ESEMeD) project. *Acta Psychiatr. Scand. Suppl.* 109 28–37. 10.1111/j.1600-0047.2004.00328.x 15128385

[B3] ^^∗^^Barth J. Munder T. Gerger H. Nüesch E. Trelle S. Znoj H. (2013). Comparative efficacy of seven psychotherapeutic interventions for patients with depression: a network meta-analysis. *PLoS Med.* 10:e1001454 10.1371/journal.pmed.1001454 PMC3665892 23723742

[B4] Barth J. Znoj H. J. Juni P. Egger R. (2007). *Revisiting the Bern Meta-analysis for Psychotherapeutic Interventions: Network Meta-analysis of Controlled Clinical Studies*. Bern: Swiss National Science Foundation.

[B5] Berardi D. Berti Ceroni G. Leggieri G. Rucci P. Ustün B. Ferrari G. (1999). Mental, physical and functional status in primary care attenders. *Int. J. Psychiatry Med.* 29 133–148. 10.2190/3D0C-QREW-1M5W-VDUU 10587811

[B6] Bergin A. E. Garfield M. J. (eds) (1994). “The effectiveness of psychotherapy,” in *The Handbook of Psychotherapy and Behaviour Change* 4th Edn (New York, NY: Wiley & Sons) 143–189.

[B7] Berk R. Freedman D. A. (2001). “Statistical assumptions as empirical commitments,” in *Law, Punishment, and Social Control: Essays in Honor of Sheldon Messinger Aldine de Gruyter* 2nd Edn eds Blomberg T. G. Cohen S. (New York, NY: Hawthorne) 235–254.

[B8] Borenstein M. Hedges L. V. Higgins J. P. T. Rothstein H. R. (2009). *Introduction to Meta-Analysis.* New York, NY: Wiley & Sons 10.1002/9780470743386

[B9] Barth J. Znoj H. J. Juni P. Egger R. (2007). *Revisiting the Bern Meta-analysis for Psychotherapeutic Interventions: Network Meta-analysis of Controlled Clinical Studies.* Bern: Swiss National Science Foundation.

[B10] Berardi D. Berti Ceroni G. Leggieri G. Rucci P. Ustün B. Ferrari G. (1999). Mental, physical and functional status in primary care attenders. *Int. J. Psychiatry Med.* 29 133–148. 10.2190/3D0C-QREW-1M5W-VDUU 10587811

[B11] Bergin A. E. Garfield M. J. (eds) (1994). “The effectiveness of psychotherapy,” in *The Handbook of Psychotherapy and Behaviour Change* 4th Edn (New York, NY: Wiley & Sons) 143–189.

[B12] Berk R. Freedman D. A. (2001). “Statistical assumptions as empirical commitments,” in *Law, Punishment, and Social Control: Essays in Honor of Sheldon Messinger Aldine de Gruyter* 2nd Edn eds Blomberg T. G. Cohen S. (New York, NY: Hawthorne) 235–254.

[B13] ^∗^Bortolotti B. Menchetti M. Bellini F. Montaguti M. B. Berardi D. (2008). Psychological interventions for major depression in primary care: a meta-analytic review of randomized controlled trials. *Gen. Hosp. Psychiatry* 30 293–302. 10.1016/j.genhosppsych.2008.04.001. 18585531

[B14] Brown C. Schulberg H. C. (1995). The efficacy of psychosocial treatments in primary care. A review of randomized clinical trials. *Gen. Hosp. Psychiatry* 17 414–424. 10.1016/0163-8343(95)00072-0 8714801

[B15] Burman E. Parker I. (eds) (1993). *Discourse Analytic Research: Repertoires and Readings of Texts in Action.* London: Routledge.

[B16] Butler A. C. Chapman J. E. Forman E. M. Beck A. T. (2006). The empirical status of cognitive-behavioural therapy: a review of meta-analyses. *Clin. Psychol. Rev.* 26 17–31. 10.1016/j.cpr.2005.07.003 16199119

[B17] Carnap R. (1928). *Der Logische Aufbau der Welt.* Leipzig: Felix Meiner Verlag.

[B18] Cohen J. (1988). *Statistical Power Analysis for the Behavioural Sciences* 2nd Edn. Hillsdale, NJ: Lawrence Erlbaum Associates.

[B19] Cooper H. Hedges L. V. (2009). “Research synthesis as a scientific process,” in *The Handbook of Research Synthesis and Meta-analysis* eds Cooper H. Hedges L. V. Valentine J. C. (New York, NY: Russel Sage) 3–16.

[B20] Cooper H. Hedges L. V. Valentine J. C. (2009). *The Handbook of Research Synthesis and Meta-analysis* 2nd Edn New York, NY: Russel Sage.

[B21] Cordray D. S. Morphy P. (2009). “Research synthesis and public policy,” in *The Handbook of Research Synthesis and Meta-analysis* eds Cooper H. Hedges L. V. Valentine J. C. (New York, NY: Russel Sage) 473–493.

[B22] Cuijpers P. Andersson G. Donker T. van Straten A. (2011b). Psychological treatment of depression: results of a series of meta-analyses. *Nord. J. Psychiatry* 65 354–364. 10.3109/08039488.2011.596570 21770842

[B23] Cuijpers P. Geraedts A. S. van Oppen P Andersson G. Markowitz J. C. van Straten A (2011a). Interpersonal psychotherapy for depression: a meta-analysis. *Am. J. Psychiatry* 168 581–592. 10.1176/appi.ajp.2010.10101411 21362740 PMC3646065

[B24] ^∗^Cuijpers P. Smit F. Bohlmeijer E. Hollon S. D. Andersson G. (2010). Efficacy of cognitive-behavioral therapy and other psychological treatments for adult depression: a meta-analytic study of publication bias. *Br. J. Psychiatry* 196 173–178. 10.1192/bjp.bp.109.066001 20194536

[B25] Cuijpers P. van Straten A. Warmerdam L. (2007a). Behavioral activation treatments of depression: a meta-analysis. *Clin. Psychol. Rev.* 27 318–326. 10.1016/j.cpr.2006.11.001 17184887

[B26] Cuijpers P. van Straten A. Warmerdam L. (2007b). Problem solving therapies for depression: a meta-analysis. *Eur. Psychiatry* 22 9–15. 10.1016/j.eurpsy.2006.11.001 17194572

[B27] Cuijpers P. van Straten A. Warmerdam L. (2008). Are individual and group treatments equally effective in the treatment of depression in adults? A meta-analysis. *Eur. J. Psychiatry* 22 38–51. 10.4321/S0213-61632008000100005

[B28] Dehue T. (2008). *De Depressie-epidemie.* Amsterdam: Augustus.

[B29] Demyttenaere K. Bruffaerts R. Posada-Villa J. Gasquet I. Kovess V. Lepine J. P. (2004). Prevalence, severity, and unmet need for treatment of mental disorders in the world health organisation. world mental health surveys. *J. Am. Med. Assoc.* 291 2581–2590. 10.1001/jama.291.21.2581 15173149

[B30] Der Wiener Kreis (1929). *Wissenschaftliche Weltauffassung Der Wiener Kreis, Veröffentlichungen des Vereines Ernst Mach, hrsg. Vom Verein Ernst Mach.* Wien: Artur Wolf Verlag.

[B31] Diggle P. J. Liang K. L. Zeger S. L. (1994). *Analysis of Longitudinal Data.* Oxford: Oxford University Press.

[B32] Dobson K. S. (1989). A meta-analysis of the efficacy of cognitive therapy for depression. *J. Consult. Clin. Psychol.* 57 414–419. 10.1037/0022-006X.57.3.414 2738214

[B33] Driessen E. Cuijpers P. de Maat SC Abbass A. A. de Jonghe F Dekker J. J. (2010). The efficacy of short-term psychodynamic psychotherapy for depression: a meta-analysis. *Clin. Psychol. Rev.* 30 25–36. 10.1016/j.cpr.2009.08.010 19766369

[B34] Ekers D. Richards D. Gilbody S. (2008). A meta-analysis of randomized trials of behavioral treatment of depression. *Psychol. Med.* 38 611–623. 10.1017/S0033291707001614 17903337

[B35] Elkin I. (1999). A major dilemma in psychotherapy outcome research: disentangling therapists from therapies. *Clin. Psychol. Sci. Pract.* 6 10–32. 10.1093/clipsy.6.1.10

[B36] Feyerabend P. (1975). *Against Method.* London: New Left Books.

[B37] Glass G. V. (1976). Primary, secondary, and meta-analysis of research. *Educ. Res.* 5 3–8.

[B38] Greenberg P. E. Kessler R. C. Birnbaum H. G. Leong S. A. Lowe S. W. Berglund P. A. (2003). The economic burden of depression in the United States: how did it change between 1990 and 2000? *J. Clin. Psychiatry* 62 1465–1475. 10.4088/JCP.v64n1211 14728109

[B39] Greenhouse J. B. Iyengar S. (2009). “Sensitivity analysis and diagnostics,” in *The Handbook of Research Synthesis and Meta-analysis* eds Cooper H. Hedges L. V. Valentine J. C. (New York, NY: Russel Sage) 417–433.

[B40] Hedges L. V. Pigott T. D. (2001). The power of statistical tests in meta-analysis. *Psychol. Methods* 6 203–217. 10.1037/1082-989X.6.3.203 11570228

[B41] Higgins J. Thompson S. Deeks J. Altman D. (2002). Statistical heterogeneity in systematic reviews of clinical trials: a critical appraisal of guidelines and practice. *J. Health Serv. Res. Policy* 7 51–61. 10.1258/1355819021927674 11822262

[B42] Higgins J. P. Green S. (eds) (2006). *Cochrane Handbook for Systematic Reviews of Interventions 4.2.6. The Cochrane Library* Issue 4 Chichester: Wiley & Sons.

[B43] Higgins J. P. Green S. (eds) (2011). *Cochrane Handbook for Systematic Reviews of Interventions Version 5.1.0 [updated March 2011]. The Cochrane Collaboration, 2011.* Available at: http://handbook.cochrane.org/

[B44] Higgins J. P. Thompson S. G. Deeks J. J. Altman D. G. (2003). Measuring inconsistency in meta-analysis. *Br. Med. J.* 327 557–560. 10.1136/bmj.327.7414.557 12958120 PMC192859

[B45] Hirschfeld R. Montgomery S. A. Keller M. B. Kasper S. Schatzberg A. F. Möller H. J. (2000). Social functioning in depression: a review. *J. Clin. Psychiatry* 61 268–275. 10.4088/JCP.v61n0405 10830147

[B46] Hunt M. (1997). *How Science Takes Stock: The Story of Meta-analysis.* New York, NY: Russell Sage.

[B47] ^∗^Huntley A. L. Araya R. Salisbury C. (2012). Group psychological therapies for depression in the community: systematic review and meta-analysis. *Br. J. Psychiatry* 200 184–190. 10.1192/bjp.bp.111.092049 22383765

[B48] Kessler R. Berglund P. Demler O. Jin R. Merikangas K. Walters E. (2005). Lifetime prevalence and age-of-onset distributions of DSM-IV disorders in the national comorbidity survey replication. *Arch. Gen. Psychiatry* 62 593–602. 10.1001/archpsyc.62.6.593 15939837

[B49] Kirsch I. (2009). *The Emperor’s New Drugs: Exploding the Antidepressant Myth.* New York, NY: Basic Books.

[B50] Knowles S. E. Toms G. Sanders C. Bee P. Lovell K. Rennick-Egglestone S. (2014). Qualitative meta-synthesis of user experience of computerised therapy for depression and anxiety. *PLoS ONE* 9:e84323 10.1371/journal.pone.0084323 PMC3894944 24465404

[B51] Kraemer H. C. Gardner C. Brooks J. O. L. Yessavage J. (1998). Advantages of excluding underpowered studies in meta-analysis: inclusionist versus exclusionist viewpoints. *Psychol. Methods* 3 23–31. 10.1037/1082-989X.3.1.23

[B52] Krumm S. Checchia C. Koesters M. Kilian R. Becker T. (2017). Men’s views on depression: a systematic review and meta-synthesis of qualitative research. *Psychopathology* 50 107–124. 10.1159/000455256 28285304

[B53] Kuhn T. (1970). *The Structure of Scientific Revolutions.* Chicago: University of Chicago Press.

[B54] Leichsenring F. (2001). Comparative effects of short-term psychodynamic psychotherapy and cognitive-behavioral therapy in depression: a meta-analytic approach. *Clin. Psychol. Rev.* 21 401–419. 10.1016/S0272-7358(99)00057-4 11288607

[B55] Light R. J. Pillemer D. B. (1984). *As cited in Cooper, H., Hedges, L. V., and Valentine, J. C. (2009). The Handbook of Research Synthesis and Meta-analysis*. New York, NY: Russel Sage.

[B56] Luborsky L. Singer B. Luborsky E. (1975). Comparative studies of psychotherapies: is it true that “Everybody has won and all must have prices?”. *Arch. Gen. Psychiatry* 32 995–1008. 10.1001/archpsyc.1975.01760260059004 239666

[B57] Madill A. Gough B. (2008). Qualitative research and its place in psychological science. *Psychol. Methods* 13 254–271. 10.1037/a0013220 18778154

[B58] Mann C. C. (1994). Can meta-analysis make policy? *Science* 266 960–962. 7973676 10.1126/science.7973676

[B59] Marecek J. (2003). “Dancing through minefields: toward a qualitative stance in psychology,” in *Qualitative Research in Psychology: Expanding Perspectives in Methodology and Design* eds Rhodes J. E. Yardley L. (Washington, DC: American Psychological Association) 49–69.

[B60] Matt G. E. Cook T. D. (2009). “Threats to the validity of generalised inferences,” in *The Handbook of Research Synthesis and Meta-analysis* eds Cooper H. Hedges L. V. Valentine J. C. (New York, NY: Russel Sage) 537–560.

[B61] Maxwell S. E. Delaney H. D. (1990). *Designing Experiments and Analysing Data.* Pacific Grove, CA: Brooks/Cole.

[B62] Miller S. D. Hubble M. A. Chow D. L. Seidel J. A. (2013). The outcome of psychotherapy: yesterday, today, and tomorrow. *Am. Psychol. Assoc. Psychother.* 50 88–97. 10.1037/a0031097 23505984

[B63] Moloney P. (2013). *The Therapy Industry: The Irresistible Rise of the Talking Cure, and Why It Doesn’t Work.* London: Pluto Press.

[B64] Moncrieff J. Churchill R. Drummond C. Mc Guire H. (2001). Development of a quality assessment instrument for trials of treatments for depression and neurosis. *Int. J. Methods Psychiatr. Res.* 10 126–133. 10.1002/mpr.108

[B65] Murray C. J. L. Lopez A. D. (1997). Alternative projections of mortality and disability by cause 1990-2020: global burden of disease study. *Lancet* 349 1498–1504. 10.1016/S0140-6736(96)07492-2 9167458

[B66] Oliver S. (1987). *As cited in Cooper, H., Hedges, L.V., & Valentine, J.C. (2009). The Handbook of Research Synthesis and Meta-analysis.* New York: Russel Sage.

[B67] Ormel J. VonKorff M. Ustun T. B. Pini S. Korten A. Oldehinkel T. (1994). Common mental disorders and disability across cultures. Results from the who collaborative study on psychological problems in general health care. *J. Am. Med. Assoc.* 272 1741–1748. 10.1001/jama.1994.03520220035028 7966922

[B68] Orwin R. G. Cordray D. S. (1985). *As cited in Cooper, H., Hedges, L.V., & Valentine, J.C. (2009). The Handbook of Research Synthesis and Meta-analysis*. New York: Russel Sage.

[B69] Orwin R. G. Vevea J. L. (2009). “Evaluating coding decisions,” in *The Handbook of Research Synthesis and Meta-analysis* eds Cooper H. Hedges L. V. Valentine J. C. (New York, NY: Russel Sage) 177–203.

[B70] Pilgrim D. Rogers A. (2010). *A Sociology of Mental Health and Illness.* Berkshire: Open University Press, McGraw-Hill Education.

[B71] Popper K. R. (1934). Logik der Forschung Wien: Springer Verlag.

[B72] Potter J. Wetherell M. (1987). *Discourse and Social Psychology.* London: Sage.

[B73] Putnam H. (1981). *Reason, Truth and History.* Cambridge: Cambridge University Press 10.1017/CBO9780511625398

[B74] Rosenthal R. (1979). The “file drawer problem” and tolerance for null results. *Psychol. Bull.* 86 638–641. 10.1037/0033-2909.86.3.638

[B75] Rosenthal R. DiMatteo M. R. (2001). Meta-analysis: recent developments in quantitative methods for literature reviews. *Annu. Rev. Psychol.* 52 59–82. 10.1146/annurev.psych.52.1.59 11148299

[B76] Rosenzweig S. (1936). Some implicit common factors in diverse methods of psychotherapy. *Am. J. Orthopsychiatry* 6 412–415. 10.1111/j.1939-0025.1936.tb05248.x

[B77] Rothstein H. R. Sutton A. J. Borenstein M. (eds) (2005). “Publication bias,” in *Publication Bias in Meta-analysis: Prevention, Assessment and Adjustment* (Hoboken, NJ: Wiley & Sons) 277–302. 10.1002/0471667196.ess7203

[B78] Shedler J. K. (2010). The efficacy of psychodynamic psychotherapy. *Am. Psychol.* 65 98–109. 10.1037/a0018378 20141265

[B79] Smith M. L. Glass G. V. Miller T. I. (1980). *The Benefits of Psychotherapy.* Baltimore, MD: The Johns Hopkins University Press.

[B80] Sterne J. A. C. Egger M. (2005). “Regression methods to detect publication and other bias,” in *Publication Bias in Meta-analysis: Prevention, Assessment and Adjustment* eds Rothstein H. R. Sutton A. J. Borenstein M. (Hoboken, NJ: Wiley & Sons.) 81–111.

[B81] Stewart W. F. Ricci J. A. Chee E. Hahn S. R. Morganstein D. (2003). Cost of lost productive work time among US workers with depression. *J. Am. Med. Assoc.* 289 3135–3144. 10.1001/jama.289.23.3135 12813119

[B82] Stiles W. B. Shapiro D. A. Elliot R. (1986). Are all psychotherapies equivalent? *Am. Psychol.* 41 165–180. 3963611 10.1037//0003-066x.41.2.165

[B83] Störig H. J. (1959/2000). Kleine Weltgeschichte der Philosophie, (transl.) P. Brommer, J. K. van den Brink, J. Thielens, Lukkenaer, P. En de Boer, and M. Geschiedenis van de filosofie. Utrecht: Het Spectrum.

[B84] Sutton A. J. (2009). “Publication bias,” in *The Handbook of Research Synthesis and Meta-analysis* eds Cooper H. Hedges L. V. Valentine J. C. (New York, NY: Russel Sage.) 435–452.

[B85] Turner E. H. Matthews A. M. Linardatos E. Tell R. A. Rosenthal R. (2008). Selective publication of antidepressant medication trials and its influence on apparent efficacy. *N. Engl. J. Med.* 358 252–260. 10.1056/NEJMsa065779 18199864

[B86] Valentine J. C. (2009). “Judging the quality of primary research,” in *The Handbook of Research Synthesis and Meta-analysis* eds Cooper H. Hedges L. V. Valentine J. C. (New York, NY: Russel Sage) 129–146.

[B87] Vanheule S. (2015). *Psychodiagnostiek ander Bekeken: Kritieken op de DSM. Een Pleidooi voor Functiegerichte Diagnostiek.* Leuven: Lannoo Campus.

[B88] Verhaeghe P. (2002). *Over Normaliteit en Andere Afwijkingen.* Leuven: Acco.

[B89] Verhaeghe P. (2005). De essentie van de psychotherapie vanuit een psychoanalytisch perspectief. *Tijdschr. Klin. Psychol.* 35 109–118.

[B90] Vermeersch E. Braeckman J. (2008). *De Rivier van Herakleitos.* Antwerpen: Houtekiet.

[B91] ^∗^Wampold B. E. Budge S. L. Laska K. M. Del Re A. C. Baardseth T. P. Fluckinger C. (2011). Evidence-based treatments for depression and anxiety versus treatment-as-usual: a meta-analysis of direct comparisons. *Clin. Psychol. Rev.* 31 1304–1312. 10.1016/j.cpr.2011.07.012 21996291

[B92] Westen D. Morrison K. (2001). A multidimensional meta-analysis of treatments for depression, panic, and generalized anxiety disorder: an empirical examination of the status of empirically supported therapies. *J. Consult. Clin. Psychol.* 69 875–899. 10.1037/0022-006X.69.6.875 11777114

[B93] WHO (2009). *Mental Health, Resilience and Inequalities.* Copenhagen: WHO Regional Office for Europe.

[B94] WHO (2012). *Depression: A Global Crisis. World Mental Health Day, October* 10 2012 Geneva: WHO.

[B95] Wittgenstein L. (1961). *Tractatus Logico-Philosophicus* (transl.) D. F. Pears and B. F. McGuinness London: Kegan Paul, Trench, Trubner & Co.

[B96] Wood W. Eagly A. H. (2009). “Advantages of certainty and uncertainty,” in *The Handbook of Research Synthesis and Meta-analysis* eds Cooper H. Hedges L. V. Valentine J. C. (New York, NY: Russel Sage) 455–472.

